# P288: The causes of disease and death of workers in the public health services from 2007 to 2011 in Guinea-Bissau

**DOI:** 10.1186/2047-2994-2-S1-P288

**Published:** 2013-06-20

**Authors:** MOA DJICÓ

**Affiliations:** 1 Sociologist, MSc, Bissau, Guinea-Bissau

## Introduction

At the end of 2007, the labor force listed in the health sector of Guiné-Bissau was 2546 employees according to data DSRHAS. In 2012 it was 1639, with 1415 active workers, 152 medium-level employees and 35 Cuban doctors. This is based on data obtained from the Finance Directorate of the Ministry of Health in the regions and health facilities where we have conducted the study.

## Objectives

Know the main causes of Morbidity, Mortality among workers in the Public Health Sector in Guinea-Bissau during the past five years (2007-2011).

## Methods

This is a qualitative study, that was conducted in all health regions of the country, particularly in the Regional Directorates of Health, National Hospital Simão Mendes National Laboratory of Public Health Centre for Mental Health, Regional Hospitals, Health Centers "A" Health Centers "B" and "C", and the Directorate of the Department of Community Health and Traditional Medicine, and the interviews were conducted with a structured questionnaire into four modules.

## Results

The study included 637 workers, 33 doctors, 396 nurses / midwives, 75 technicians,, 24 pharmacists / pharmacy technicians, administrative staff 78/29 officials and other different professions totaling 244, corresponding to 39, 19% of men and 379 women equivalent to 60.84%. Causes of death were reported for 59 workers. According Verbal Autopsy (VA) we identified five major diseases: hypertension 27%, HIV 22% , Diabetes 94%; Tuberculosis 15.25% Hepatitis 6.77% and the rest 12.04%.

## Conclusion

Precarious working conditions of workers, focusing on the status of biosecurity in the workplace. Most professionals believe that it is not well taken care of by employers, in this case, the Guinean government, because it does not fund medical and pharmacological actions. The high mortality rate seems to stem largely from working conditions where the employee is constantly at risk.

## Competing interests

None declared

